# Variation in antenatal anaemia guidelines across the NHS in the United Kingdom: A cross‐sectional study

**DOI:** 10.1111/bjh.70780

**Published:** 2026-08-20

**Authors:** Sarah Haynes, Islam Balboula, Peter Collins, William Parry‐Smith, Haddy Fye, Amy Elsmore, Rachel Collis, Lucy De Lloyd, David Churchill, Sarah F. Bell, Simon J. Stanworth

**Affiliations:** ^1^ Nuffield Department of Women's and Reproductive Health University of Oxford Oxford UK; ^2^ Department of Anaesthesia and Critical Care Medicine Cardiff and Vale University Health Board Cardiff UK; ^3^ Institute of Infection and Immunity, School of Medicine Cardiff University Cardiff UK; ^4^ Department of Obstetrics and Gynaecology The Shrewsbury and Telford Hospital NHS Trust Shrewsbury UK; ^5^ Keele University School of Medicine Keele UK; ^6^ Patient and Public Involvement and Engagement Representative, OBS‐UK Trial UK; ^7^ Department of Obstetrics The Royal Wolverhampton NHS Trust, New Cross Hospital Wolverhampton UK; ^8^ Research Institute for Healthcare Science University of Wolverhampton Wolverhampton UK; ^9^ Department of Haematology NHS Blood and Transplant Oxford UK; ^10^ Radcliffe Department of Medicine University of Oxford Oxford UK

**Keywords:** anaemia, antenatal, guidelines, iron deficiency, NHS, pregnancy

## Abstract

Antenatal anaemia affects up to 30% of pregnant women in the United Kingdom and is a recognised clinical priority. National management guidance is published by the British Society of Haematology (BSH), but the extent to which local National Health Service (NHS) guidelines align with it is unknown. We conducted a cross‐sectional document analysis of antenatal anaemia guidelines from 49 NHS sites across the United Kingdom, obtained through two national research programmes. Two reviewers independently extracted data on screening, diagnosis, treatment and monitoring, using the 2020 BSH guideline as the national reference standard. The included guidelines represented approximately 33% of annual UK births. Screening and diagnostic haemoglobin thresholds were broadly consistent with BSH guidance, whereas substantial variation was concentrated in treatment and monitoring: oral iron indications, dosing and duration; haemoglobin thresholds and clinical criteria for intravenous iron; and the timing, biomarkers and response definitions used after treatment. Ferritin thresholds, reassessment intervals and response criteria were frequently absent or divergent. Variation was greatest in domains where the underlying evidence base is least resolved. These findings characterise the nature and extent of guideline‐level variation across NHS maternity care and identify priority areas for future research and national guideline development.

## INTRODUCTION

Iron deficiency anaemia (IDA) is the most common cause of anaemia in pregnancy, estimated to affect up to 30% of pregnant women in the United Kingdom.^1^, ^2^ Even in the absence of anaemia, maternal iron deficiency is associated with fatigue, reduced quality of life and impaired cognitive and physical function.^3^ IDA is associated with additional risks, including postpartum haemorrhage, blood transfusion, preterm birth and low birthweight.^4^ Early identification and treatment are therefore key components of good antenatal care,^5^, ^6^ and the British Society of Haematology (BSH) publishes national guidelines to support evidence‐based management across UK maternity services, with the most recent update in 2020.^7^

Despite this national framework, variation in clinical practice remains a recognised challenge. A recent UK‐based audit found that adherence to BSH guidance for antenatal anaemia management was variable and frequently suboptimal,^2^ raising questions about the factors driving inconsistency in care. In the United Kingdom, local National Health Service (NHS) trusts and health boards responsible for delivering maternity services across England, Scotland, Wales, and Northern Ireland (referred to collectively as ‘NHS sites’ throughout this manuscript) develop their own clinical guidelines, adapting national recommendations to local populations and resources.^8^, ^9^ The extent to which these local guidelines align with BSH national recommendations has not previously been evaluated at scale.

IDA in pregnancy has been an area of considerable and growing research interest, with the evidence base continuing to evolve across multiple aspects of clinical management. Variation in guideline recommendations has been demonstrated internationally, with a recent systematic review and quality appraisal of 16 international guidelines identifying substantial differences in diagnostic thresholds, treatment recommendations and monitoring strategies (see Table S1).^10^ Keeping local guidance current with an evolving evidence base represents an ongoing challenge, and unwarranted variation in clinical practice is recognised as a challenge for healthcare quality, safety and equity,^11^ with addressing it identified as a priority in the NHS 2025/26 operational planning guidance.^12^ This study aimed to evaluate local NHS antenatal anaemia guidelines and compare them with current BSH national recommendations, to characterise the nature and extent of variation across NHS sites in the United Kingdom.

## METHODS

### Study design

This was a cross‐sectional, document‐based analysis of antenatal anaemia guidelines in use across a national sample of NHS sites, with comparison to national guidance. A predefined extraction framework and independent dual review were used to support structured and reproducible data extraction. The study was conducted as part of two broader research programmes: the *Obstetric Bleeding Study UK* (OBS UK) and the *Primary prevention of maternal Anaemia to avoid preterm Delivery and other Adverse Outcomes* (PANDA) trial. OBS UK is a nationwide initiative evaluating a standardised approach to the recognition and management of postpartum haemorrhage across 36 NHS sites in the United Kingdom. PANDA is a large‐scale trial investigating the effectiveness of routine iron supplementation for the prevention of antenatal anaemia.

The 2020 BSH guideline was selected as the national reference standard because it remains the current UK guideline specifically addressing iron deficiency and anaemia in pregnancy; other relevant UK guidance, including RCOG,^13^ is more transfusion‐focused and refers to BSH in this area. BSH guidance was used as a structured comparator rather than as an assumption of definitive best practice, recognising that the evidence base has evolved since its publication and that alternative recommendations may reflect other evidence or international guidance.

### Data collection

Local guidelines were obtained through email requests sent to participating NHS sites in both studies between September 2024 and June 2025. To ensure regional representation, one site (not part of OBS UK or PANDA) was also approached via email. All responding sites consented to provide their anonymised guidelines for research purposes. For sites that provided incomplete or unclear guidelines, follow‐up requests were made to obtain further clarification and ensure the data were complete. Two guidelines were excluded due to incomplete or unclear guidelines (both one‐page flow diagrams with no further information). Four duplicates were removed (four sites were participating in both studies). Additionally, publicly available anaemia guidelines from three additional Welsh sites were obtained via the WISDOM NHS Wales website, without direct contact with these sites. Data collection was completed in June 2025. As many local NHS guidelines are not systematically indexed or publicly available, updates issued after this date could not be comprehensively identified across all included sites. Some sites may therefore have subsequently revised their guidance, and the analysis should be interpreted as a cross‐sectional snapshot of local guidance available during the data collection period.

### Data extraction

A structured data extraction form (see Table S2) was used to ensure standardised collection of data across all guidelines. Two independent reviewers extracted information on the core outcomes:

- Screening recommendations (haemoglobin and ferritin testing, routine vs. risk‐based screening).
- Diagnostic thresholds for anaemia and iron deficiency.
- Oral iron treatment indications, formulations and dosage.
- Intravenous (IV) iron initiation thresholds and preferred formulations.
- Post‐treatment monitoring intervals and biomarkers.

In UK maternity care, ‘booking’ refers to the first formal antenatal visit, typically occurring in the first trimester. Missing information was recorded as ‘not stated’. Each reviewer cross‐checked the other's extracted data, with disagreements resolved through discussion. Alignment was assessed separately for each domain by comparing local recommendations with the corresponding BSH reference standard. Guidelines were classified as ‘aligned’ if recommendations matched BSH guidance, ‘aligned with additional measures’ if they met core BSH recommendations but included additional non‐conflicting measures, ‘not aligned’ if they diverged from BSH guidance, or ‘not stated’ if no relevant recommendation was provided. Classification as ‘not aligned’ indicates divergence from the BSH reference standard and does not imply that a local recommendation is incorrect or of lower quality; differences may reflect alternative evidence, expert consensus or other national or international guidance. Analyses and data visualisations were performed using RStudio (version 2024.12.0 + 467), with descriptive statistics used to characterise variation across NHS site guidelines.

## RESULTS

### Characteristics of NHS sites

**FIGURE 1:**
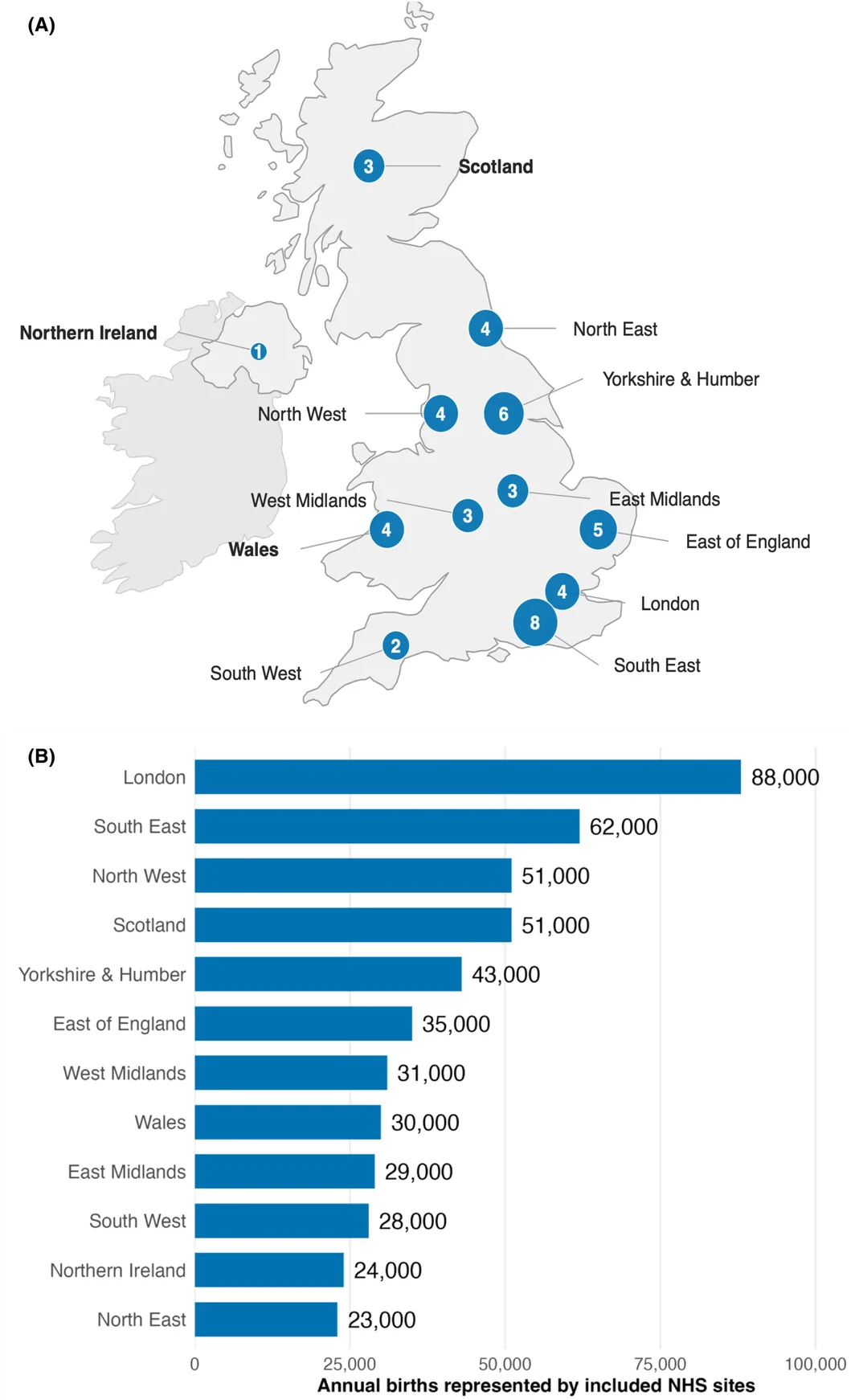
Geographical distribution of included NHS site guidelines and annual births represented across UK regions. (A) Number of local antenatal anaemia guidelines included from each UK region (*n* = 49). (B) Estimated annual births represented by the included NHS sites within each region, derived from publicly available national statistics. Together, the included sites represent approximately 216 000 births annually, around one‐third of UK births. NHS, National Health Service.

A total of 49 NHS Sites guidelines were included, with representation from England (*n* = 41), Scotland (*n* = 3), Wales (n = 4) and Northern Ireland (*n* = 1). Sixteen NHS sites provided guidelines through PANDA, and 36 NHS sites provided guidelines through OBS UK. The combined annual number of births across the 49 sites included was 216 449, approximately 33% of the United Kingdom's annual birth rate^14^, ^15^, ^16^, ^17^, ^18^, ^19^ (see Figure 1).

The median publication date of the guidelines was March 2023 (range: December 2010 to March 2025), with a median expiry date of November 2025 (range: November 2015 to August 2028). The median duration of guideline validity was 3 years. The majority (*n* = 43) of guidelines were published after the updated national guidelines were published in early 2020. The general adherence of local site guidelines to national guidelines is summarised in Table 1.

**TABLE 1:** Summary of general alignment with national guidelines by NHS sites (*n* = 49).

Domain	Aligned	Aligned with additional measures	Not aligned	Not stated
*Routine screening*				
Hb (Booking^a^ and 28 weeks)	41 (84%)	8 (16%)	0 (0%)	0 (0%)
Ferritin	41 (84%)	8 (16%)	0 (0%)	0 (0%)
*Diagnosis*				
Anaemia	47 (96%)	0 (0%)	2 (4%)	0 (0%)
Iron deficiency	31 (63%)	0 (0%)	3 (6%)	15 (31%)
*Oral iron treatment*				
Indication	33 (67%)	3 (6%)	5 (10%)	8 (16%)
Preparation	46 (94%)	0 (0%)	0 (0%)	2 (4%)
Dose	34 (69%)	0 (0%)	13 (27%)	2 (4%)
Length of Treatment	10 (20%)	4 (8%)	18 (37%)	17 (35%)
*IV iron treatment*				
Indication	4 (8%)	30 (61%)	0 (0%)	15 (31%)
Preparation	43 (88%)	0 (0%)	1 (2%)	5 (10%)
*Monitoring post oral iron*				
Biomarker	32 (65%)	15 (31%)	0 (0%)	2 (4%)
Timing	31 (63%)	6 (12%)	10 (20%)	2 (4%)
Response definition	16 (33%)	16 (33%)	0 (0%)	17 (35%)

*Note*: Each local guideline recommendation was assessed against the corresponding 2020 BSH national guideline recommendation and classified as aligned, aligned with additional measures, not aligned or not stated.

Abbreviations: BSH, British Society for Haematology; NHS, National Health Service.

^a^ Booking refers to the first antenatal visit, typically in the first trimester.

### Screening practices

Hb screening was universally recommended at booking and at 28 weeks, consistent with the national guidelines. Seven guidelines recommend an additional routine Hb test at 32–36 weeks for all pregnant people. This is only recommended for twin pregnancies in the national guidelines.

Ferritin screening guidelines were universally aligned with national recommendations. In addition, eight local guidelines (16%) advised routine ferritin screening at booking for all pregnant people, with seven of these also recommending a repeat screening at 28 weeks. National guidelines state that there is insufficient evidence to support routine ferritin screening for all pregnancies, instead recommending it only for women with a known haemoglobinopathy or identified risk factors for iron deficiency.

### Diagnostic criteria

Trimester‐based Hb diagnostic thresholds for anaemia were consistent except for two recently published guidelines (2023–2025), which used a Hb <120 g/L for the duration of pregnancy to define anaemia.

Ferritin thresholds for diagnosing iron deficiency were largely consistent with 31 guidelines (63%) using the <30 μg/L threshold recommended by the national guidelines. Ferritin thresholds were not stated in 14 guidelines (29%). One guideline, published in 2022, used a threshold of <50 μg/L and another used <30 μg/L for the first trimester and <15 μg/L after the first trimester, with another using <15 μg/L for the duration of the pregnancy.

### Oral iron treatment

Indications for oral iron treatment were varied; national guidelines recommend starting oral iron for any pregnant person with anaemia (1st trimester: Hb <110 g/L, 2nd trimester onwards: Hb <105 g/L) or risk factors for IDA. The indications used by local sites are summarised in Table S3.

**FIGURE 2:**
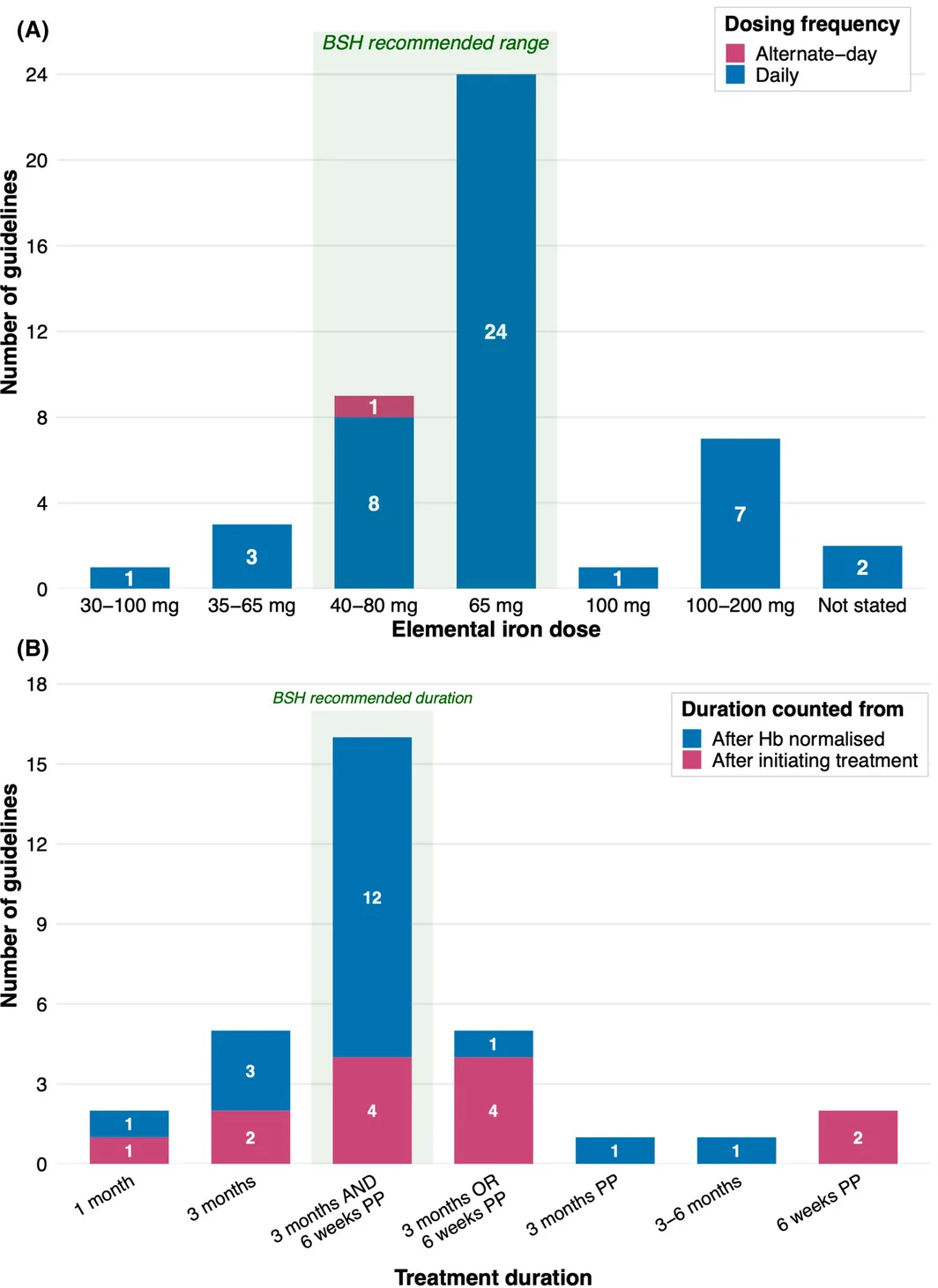
Local NHS site guidelines' recommendations for oral iron therapy in pregnancy. (A) Recommended elemental iron dose and dosing frequency. (B) Recommended duration of treatment, and whether duration was calculated from treatment initiation or from haemoglobin normalisation. Green shaded regions indicate the recommendations of the 2020 BSH guideline. BSH, British Society for Haematology; NHS, National Health Service; PP, postpartum.

Oral iron formulations were consistent, with most recommending ferrous sulphate or ferrous fumarate as first line. Variability was noted in oral iron dosing recommendations. National guidelines recommend daily dosing of 40–80 mg of elemental iron with the option to change to alternate daily dosing to reduce gastrointestinal side effects. Elemental iron dosages recommended by local sites are shown in Figure 2A.

### Intravenous iron treatment

Forty (82%) guidelines recommended either ferric carboxymaltose (Ferrinject) or ferric derisomaltose (Monofer), aligning with national guideline recommendations. However, despite national guidance stating that there is insufficient evidence to support the use of iron hydroxide dextran (Cosmofer) in pregnancy, one guideline (published in 2012) recommended Cosmofer as a first‐line option and another (published in 2023) listed it as an alternative to ferric carboxymaltose.

Local NHS site recommendations for haemoglobin thresholds to initiate IV iron varied from <65 g/L to <110 g/L (Figure S1). Some guidelines stratified thresholds based on symptoms, using lower cut‐offs for asymptomatic individuals. Specific Hb thresholds were often used alongside other clinical criteria such as non‐compliance, intolerance, failure to respond, malabsorption or symptomatic (summarised in Table S4). This suggests that IV iron was generally not recommended as first‐line treatment. However, in guidelines that reference Hb thresholds in late gestation, IV iron appeared to be recommended without clear instruction to trial oral iron first.

### Monitoring response to iron treatment

Local NHS site guidelines varied significantly in their recommendations for monitoring response to both oral and IV iron treatment.

**FIGURE 3:**
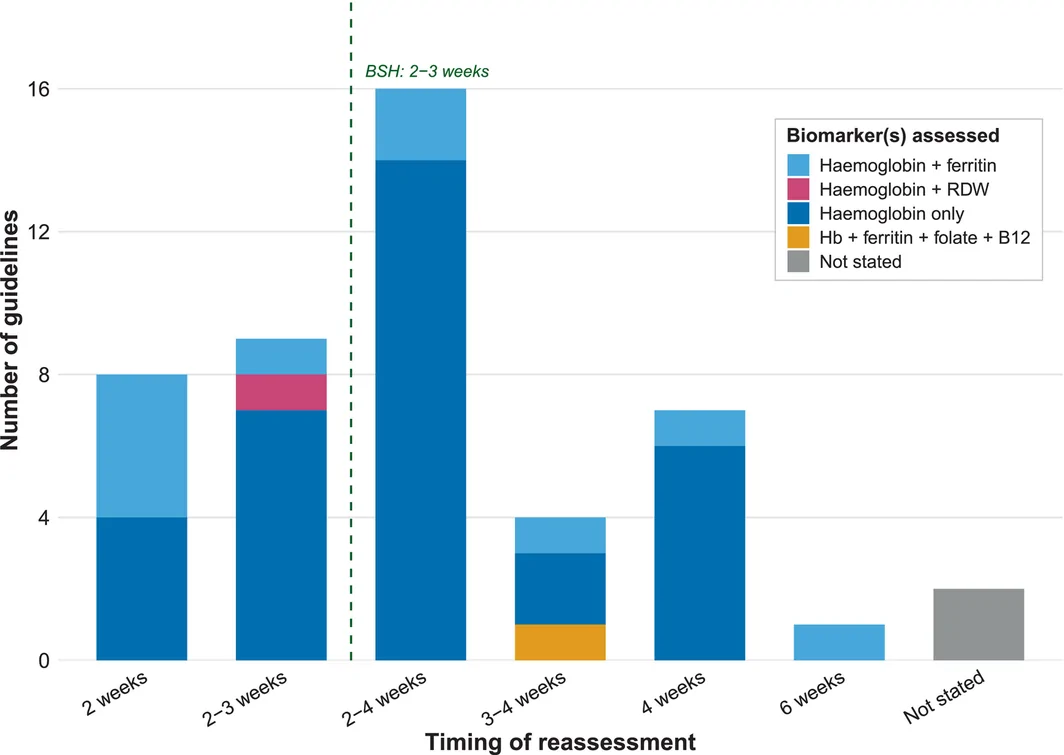
Recommendations for monitoring response to oral iron treatment. Timing of reassessment and the biomarker(s) recommended for monitoring response to oral iron therapy. The dashed line indicates the timing recommended by the 2020 BSH guideline (2–3 weeks). BSH, British Society for Haematology.

For oral iron, Hb testing alone was used by 32 (65%) of the guidelines to assess response; however, reassessment intervals ranged from 2 to 6 weeks. The variation in timing and tests used to monitor response is shown in Figure 3.

Response to oral treatment was inconsistently defined. Sixteen guidelines (33%) defined response as any increase in haemoglobin, consistent with the general BSH recommendation of a ‘demonstrable rise’ without a specified numeric threshold; a further 15 guidelines (31%) specified a numeric Hb increase (ranging from 10 g/L over 1 week to 20 g/L over 4–6 weeks) and one guideline used an increase in red cell distribution width (RDW), and 17 (35%) provided no guidance (see Table S5).

For IV iron, 31 (63%) guidelines included monitoring recommendations. All 31 guidelines advised repeat testing with Hb testing, with a smaller number including ferritin (6 guidelines) or reticulocyte count (2 guidelines). The most common follow‐up interval was 2–4 weeks (18 guidelines). Only four guidelines defined a treatment response. Comparison with national guidance is limited, as national guidelines do not currently provide recommendations on monitoring response to IV iron.

## DISCUSSION

This study provides a national evaluation of antenatal anaemia guideline recommendations across NHS sites and, to our knowledge, is the first to examine the implementation of BSH antenatal anaemia guidance at the level of local NHS written guidance. Variation was not uniformly distributed across domains of care. While screening and diagnostic haemoglobin thresholds were largely consistent, substantial variation was concentrated in treatment and monitoring, particularly where national guidance is limited or the evidence base remains uncertain. These findings are consistent with previous research demonstrating variability in anaemia management within the United Kingdom and internationally,^2^, ^10^ but extend this work by showing that inconsistency may arise not only from individual clinical practice but also from variation in the local written guidance clinicians follow. While gaps between guidelines and clinical practice are well documented,^20^, ^21^, ^22^ this study identifies variation upstream of clinical practice, with implications for efforts to improve consistency and equity in antenatal anaemia care.

The causes of variation are likely multifactorial. Not all variation is necessarily unwarranted: some local adaptation may appropriately reflect differences in population need, appointment scheduling, workforce capacity and the competing clinical demands of providing maternity care.^23^ Furthermore, BSH recommendations are not uniformly supported by high‐certainty evidence and differ in some areas from international guidance.^7^ Divergence from BSH should therefore not necessarily be interpreted as inappropriate local guidance, as some recommendations may reflect alternative evidence, expert consensus, or other national or international guidance (Table S1). Nevertheless, variation was greatest in areas where the evidence base remains least resolved. Uncertainty regarding oral iron dosing,^7^, ^24^, ^25^, ^26^ intravenous iron treatment^27^ and post‐treatment monitoring^28^ is well recognised, while international guidelines also differ across these domains.^10^ The suboptimal adherence to BSH guidance identified in the Churchill et al. national audit^2^ may therefore partly reflect the guideline‐level variation identified here.

Specific areas of practice uncertainty were highlighted by our analysis. Prophylactic oral iron is not routinely recommended in UK national guidelines due to limited evidence of clinical benefit,^7^ inconclusive potential associations with outcomes such as pre‐eclampsia^29^ and gestational diabetes,^30^ and concerns around providing treatment that can be associated with unpleasant side effects to women who may not need it. For treatment of anaemia with oral iron, most local guidelines aligned with BSH dosing recommendations, although some continued to recommend higher daily doses of 100–200 mg, consistent with older practice rather than more recent evidence favouring lower doses for improved tolerance and fractional absorption.^7^, ^24^, ^25^ Treatment duration was frequently unspecified, while recommendations for monitoring response varied in both timing and definition. Although BSH recommends haemoglobin reassessment at 2–3 weeks, it does not specify a pregnancy‐specific numeric threshold for adequate response. The observed variation may, therefore, reflect limited consensus on how treatment response should be defined during pregnancy, despite its importance for subsequent treatment escalation. In a cohort of over 20 000 pregnant women, 17.8% were refractory to oral iron supplementation, with poor response associated with adverse pregnancy outcomes.^31^ Consistent monitoring guidance is, therefore, essential to support timely identification of non‐response and clinical decision‐making around escalation.

Substantial variation was also observed in thresholds for intravenous iron. IV iron produces a more rapid haematological response than oral iron, although evidence for improvement in maternal and neonatal clinical outcomes has historically been less consistent.^32^ More recently, a multicentre randomised trial in women with non‐anaemic iron deficiency reported higher mean birthweight with IV iron than usual care, although birthweight was a secondary outcome, the trial was not powered for clinical end‐points, and this population differs from the anaemic women who are the primary focus of the present study.^33^ Alongside this evolving evidence base, the greater cost and service requirements associated with parenteral treatment may further complicate decisions regarding IV iron use.^32^ A survey of Australian and New Zealand obstetricians similarly demonstrated substantial variation in IV iron practice.^34^ Together, these findings highlight the complexity of developing consistent recommendations for IV iron use and an important area for future evidence synthesis and guideline development.

The direct impact of the guideline variation identified here remains uncertain. As a document‐based study, this analysis cannot establish whether differences in written recommendations translate into differences in care or patient outcomes. Nevertheless, the findings have practical relevance for clinicians, guideline developers and researchers.

For clinicians, the findings serve as a reminder that local guidelines may not always reflect the most current evidence, particularly in areas where the evidence base is evolving. In the domains where the greatest variation was observed, including oral iron dosing, treatment response monitoring and IV iron escalation criteria, local guidance may differ from BSH recommendations for a range of reasons, including local adaptation and the use of alternative or emerging evidence. In these areas, familiarity with current national guidance and engagement with the emerging evidence base may support more consistent clinical decision‐making.

For guideline developers, nationally and locally, these same domains reflect areas where the evidence base remains insufficiently resolved to support clear, consistent recommendations. Addressing unwarranted variation is a stated NHS priority,^12^ yet developing and maintaining local guidelines requires substantial time and resources.^22^ Where national guidance is necessarily non‐specific due to limited evidence, consistent local implementation becomes difficult, underscoring the importance of prioritising research in these areas to support future guideline development.

For researchers, two priorities emerge. First, studies linking guideline content to prescribing behaviour and patient outcomes are needed to determine whether the variation identified here has consequences for the quality and equity of antenatal anaemia care. Second, and more fundamentally, resolving the underlying evidence gaps in oral iron prophylaxis and dosing, treatment response monitoring and IV iron escalation criteria is essential if future national guidance is to provide the specificity that local implementation requires.

This study has strengths and limitations. The sample was large and geographically diverse, covering approximately 33% of annual UK births across 49 NHS sites, with structured extraction and independent dual review. However, sites were drawn primarily from two national research programmes rather than a random or exhaustive sample of UK NHS sites, and selection bias cannot be excluded. Real‐world adherence to local guidance was not assessed, and practice may differ from written recommendations. Some sites may also have revised their guidance after data collection ended in June 2025. The use of the 2020 BSH guideline as the reference standard is a further limitation. Although it remains the current UK guideline specifically addressing iron deficiency and anaemia in pregnancy, some included local guidelines predated its publication, while the evidence base continued to evolve during the period covered by this study, particularly for oral iron dosing and IV iron.^26^, ^27^ Some divergence among more recent local guidelines may therefore reflect evolving evidence rather than delayed implementation. Data on haemoglobinopathy‐specific recommendations were not extracted and warrant further study. Finally, this study characterises variation but cannot establish its impact on clinical outcomes.

## CONCLUSIONS

This study provides the first national analysis of antenatal anaemia guideline recommendations across NHS sites in the United Kingdom, identifying consistent screening practices but substantial variation in treatment and monitoring. Variation was greatest where the evidence base remains least resolved, including oral iron dosing, intravenous iron escalation and post‐treatment monitoring. These findings identify priority areas for future research and guideline development and raise questions about whether variation in local guidance contributes to differences in clinical practice and equity of care across NHS settings.

## AUTHOR CONTRIBUTIONS

**Peter Collins:** Investigation; writing – review and editing; project administration. **Sarah Haynes:** Conceptualization; methodology; validation; visualization; writing – review and editing; writing – original draft; investigation; data curation; formal analysis. **Amy Elsmore:** Writing – review and editing; project administration. **Lucy De Lloyd:** Writing – review and editing; project administration. **Sarah F. Bell:** Supervision; funding acquisition; writing – review and editing; project administration; investigation; conceptualization; methodology. **Rachel Collis:** Writing – review and editing; project administration. **Islam Balboula:** Validation; investigation; data curation; writing – review and editing. **Simon J. Stanworth:** Conceptualization; investigation; funding acquisition; writing – review and editing; project administration; supervision; methodology. **William Parry‐Smith:** Investigation; writing – review and editing; project administration. **David Churchill:** Writing – review and editing; conceptualization; funding acquisition; investigation; supervision; methodology; project administration. **Haddy Fye:** Writing – review and editing.

## CONFLICT OF INTEREST STATEMENT

S.B., R.C., L.D.L. and P.C. have received research support from Haemonetics, Werfen and CSL Behring. S.B., R.C. and P.C. declare a paid consultancy for CSL Behring. RC and PC declare a paid consultancy, and SB has received lecture honoraria from Werfen. There are no other conflicts of interest declared by other authors.

## ETHICS STATEMENT

The OBS UK study and the PANDA study both received Health Research Authority (HRA) approval (OBS UK: REC/NW/0242, approved 21/09/2023; PANDA: REC 23/NS/0123, approved 17/11/2023; MHRA CTA 25224/0009/001‐000, approved 27/11/2023). No further ethics approval was required for this document‐based evaluation, as no patient‐identifiable data were used and all participating NHS sites were anonymised in the reporting of results.

## PATIENT CONSENT STATEMENT

Not applicable. This study did not involve any individual patient data; it is a document‐based analysis of publicly and institutionally provided NHS guideline documents.

## PERMISSION TO REPRODUCE MATERIAL FROM OTHER SOURCES

Not applicable. No copyrighted material from third‐party sources has been reproduced in this manuscript.

## CLINICAL TRIAL REGISTRATION

Not applicable. This is a document‐based, descriptive cross‐sectional study and is not a clinical trial. (The contributing PANDA trial is registered separately; see MHRA CTA 25224/0009/001‐000 cited above.)

## Supporting information


**Table S1.** Selected national and international recommendations for the assessment and management of iron deficiency and anaemia in pregnancy.
**Table S2.** Data extraction tool with BSH National Guidelines as exemplar.
**Table S3.** Local NHS site guidelines' indications for oral iron by trimester.
**Figure S1.** Haemoglobin thresholds used by local NHS site guidelines to indicate IV iron treatment.
**Table S4.** Local NHS site recommendations for IV iron indications.
**Table S5.** Local NHS site guidelines' definitions of adequate response to oral iron treatment.

## Data Availability

The datasets used and/or analysed during the current study are available from the corresponding author on reasonable request.
