# Supplementary material for: Variation in antenatal anaemia guidelines across the NHS in the United Kingdom: A cross‐sectional study
Source: Br J Haematol. 2026 Aug 20;209(3):1301–10. doi: 10.1111/bjh.70780 (PMC13570162; doi:10.1111/bjh.70780)
Supplement: Supplementary file 1 [file BJH-209-1301-s001.docx]

**Supplementary Tables and Figures:**

**Supplementary Table S1.** Selected national and international recommendations for the assessment and management of iron deficiency and anaemia in pregnancy

| **Domain** | **BSH 2020**^1^ | **HOW 2025**^2^ | **WHO**^3,4^ **2020** | **FIGO 2025**^5^ | **ACOG 2021**^6^ |
| --- | --- | --- | --- | --- | --- |
| **Routine Screening** | | | | | |
| **Hb** | Booking and 28 weeks; additional screening for high-risk pregnancies. | FBC at booking and 24–28 weeks. | Hb assessment as part of routine antenatal care. | Booking and 28 weeks. | FBC in first trimester and 24–28 weeks. |
| **Ferritin** | Risk-based testing for women with known haemoglobinopathy or risk factors for iron deficiency. | Ferritin at booking and 24–28 weeks; repeat at 36 weeks if clinically indicated. | Not routinely recommended in ANC guidance. | Where iron deficiency is suspected. | Not routinely recommended; used in evaluation of suspected iron deficiency. |
| **Diagnosis** | | | | | |
| **Anaemia** | Hb <110 g/L first trimester; <105 g/L second/third trimester. | Not anaemia-specific; focuses on iron deficiency. | Hb <110 g/L first/third trimester; <105 g/L second trimester. | Hb <110 g/L all trimesters. | Hb <110 g/L first/third trimester; <105 g/L second trimester. |
| **Iron Deficiency** | SF <30 µg/L. | SF <30 µg/L. | SF <15 µg/L. | SF <30 µg/L. | SF <30 µg/L |
| **Oral Iron Treatment** | | | | | |
| **Indication** | Anaemia; prophylactic iron may be considered for women at increased risk of iron deficiency. | Iron deficiency or IDA. | Prophylactic iron for all pregnant women; treatment dose if anaemia is diagnosed. | Prophylactic iron for all pregnant women; therapeutic oral iron for IDA. | Low-dose supplementation in pregnancy; oral iron first-line for IDA. |
| **Dose** | 40–80 mg elemental iron daily. | No single universal dose specified; alternate-day dosing may be considered to limit side effects. | 30–60 mg elemental iron daily for prophylaxis; 120 mg daily if anaemia is diagnosed. | 30–60 mg elemental iron daily for prevention; 60–120 mg daily for IDA. | No single therapeutic elemental-iron dose specified in the recommendation wording summarised here. |
| **IV Iron Treatment** | | | | | |
| **Indication** | For confirmed IDA with intolerance or inadequate response to oral iron. | ID or IDA with intolerance or refractoriness to oral iron, or in the third trimester. | Not specified | Malabsorption/intolerance or non-response to oral iron, or severe iron deficiency later in pregnancy. | Intolerance or non-response to oral iron, or severe iron deficiency later in pregnancy. |
| **Monitoring Post Oral Iron Treatment** | | | | | |
| **Biomarker** | Hb. | Not specified. | Not specified. | Hb. | Hb/Hct. |
| **Timing** | 2–3 weeks. | Not specified. | Not specified. | 2 weeks. | Reticulocytosis at 7–10 days, followed by Hb/Hct rise in subsequent weeks. |
| **Response** | Demonstrable Hb rise; no specific numeric threshold. | Not specified. | Not specified. | Hb rise ≥10 g/L after 2 weeks. | Not specified. |

Guidance was selected to contextualise the BSH reference standard across complementary clinical perspectives: UK national haematology guidance, recent international consensus focused on iron deficiency, global public health guidance, international obstetric consensus, and national obstetric guidance from another high-income healthcare setting. The comparison is illustrative rather than exhaustive and was not used to assess alignment of local NHS guidelines.

***Abbreviations:*** *ACOG, American College of Obstetricians and Gynecologists; ANC, antenatal care; BSH, British Society for Haematology; CBC, complete blood count; FBC, full blood count; FIGO, International Federation of Gynecology and Obstetrics; Hb, haemoglobin; Hct, haematocrit; HOW, Haematology in Obstetric and Women’s Health; ID, iron deficiency; IDA, iron deficiency anaemia; IV, intravenous; SF, serum ferritin; WHO, World Health Organization*

***Supplementary References:***

1.	Pavord S, Daru J, Prasannan N, Robinson S, Stanworth S, Girling J, et al. UK guidelines on the management of iron deficiency in pregnancy. British Journal of Haematology [Internet]. 2020 [cited 2023 Nov 28]. Available from: https://qmro.qmul.ac.uk/xmlui/bitstream/handle/123456789/59409/Pavord%20et%20al.%202012.%20UK%20guideline.docx?sequence=2

2.	Clarke L, Froessler B, Tang C, King K, Ross B, Kidson‐Gerber G, et al. Iron optimisation in pregnancy: a Haematology in Obstetric and Women’s Health Collaborative consensus statement. Intern Med J. 2025 Feb;55(2):300–7. doi:10.1111/imj.16602 PubMed PMID: 39907166; PubMed Central PMCID: PMC11817901.

3.	World Health Organization. WHO antenatal care recommendations for a positive pregnancy experience. Nutritional interventions update: Multiple micronutrient supplements during pregnancy. World Health Organization; 2020.

4.	World Health Organization. WHO guideline on use of ferritin concentrations to assess iron status in individuals and populations [Internet]. Geneva: World Health Organization; 2020 [cited 2024 Mar 28]. Available from: https://iris.who.int/handle/10665/331505

5.	Ubom AE, Begum F, Ramasauskaite D, Nieto-Calvache AJ, Oguttu M, Nunes I, et al. FIGO good practice recommendations on anemia in pregnancy, to reduce the incidence and impact of postpartum hemorrhage (PPH). Int J Gynaecol Obstet. 2025 Dec;171(3):993–1007. doi:10.1002/ijgo.70529 PubMed PMID: 41031541; PubMed Central PMCID: PMC12640178.

6.	Gynecologists OAc. Anemia in pregnancy: ACOG practice bulletin, number 233. Obstetrics and gynecology. 2021;138(2).

## **Supplementary Table S2:** Data Extraction Tool with BSH National Guidelines as Exemplar

The data extraction tool comprised six domains, each corresponding to a key area of antenatal anaemia management. Below, the tool structure is presented with data extracted from the BSH national guidelines (2020) as an illustrative example.

| **BSH Guidelines on Management of Iron Deficiency in Pregnancy (2019) — Summary** | | |
| --- | --- | --- |
| **Category** | **Detail** | |
| Guideline Name | UK Guidelines on the Management of Iron Deficiency in Pregnancy (BSH) | |
| Date Published | October 2019 | |
| Expiry / Review Date | Not specified | |
| **A. Haemoglobin Screening** | | |
| **Timepoint** | **Recommended** | **BSH Recommendation** |
| Booking | Yes | Yes |
| 28 Weeks | Yes | Yes |
| 32–36 Weeks | No | No (twin pregnancies only) |
| **B. Serum Ferritin Screening** | | |
| **Timepoint** | **Recommended** | **BSH Recommendation** |
| Booking | No | No (risk-based only) |
| 28 Weeks | No | No (risk-based only) |
| Indication for Testing | Women at risk of iron deficiency OR haemoglobinopathy | |
| **C. Diagnostic Criteria** | | |
| **Parameter** | **BSH Threshold** | |
| 1st Trimester Anaemia | Hb <110 g/L | |
| 2nd & 3rd Trimester Anaemia | Hb <105 g/L | |
| Postpartum Anaemia | Hb <100 g/L | |
| Iron Deficiency | Serum ferritin <30 µg/L | |
| **D. Oral Iron Treatment** | | |
| **Parameter** | **BSH Recommendation** | |
| Indication | Hb <110 g/L (1st trimester); Hb <105 g/L (2nd or 3rd trimester); At-risk women (prophylactic) | |
| Preparation | Ferrous fumarate or equivalent iron salts | |
| Dose | 40–80 mg elemental iron daily; alternate-day dosing permitted to reduce GI side effects | |
| Duration | 3 months after Hb normalised and at least 6 weeks postpartum | |
| **E. Intravenous Iron Treatment** | | |
| **Parameter** | **BSH Recommendation** | |
| **Indications** | | |
| Hb Threshold | Hb <70 g/L | |
| Timing Threshold | Anaemic at >34 weeks gestation | |
| Failure of Oral Iron | Intolerance or lack of response to oral iron | |
| 1st Trimester Use | Not recommended | |
| Non-iron Deficiency Anaemia | Not indicated | |
| **Preparation & Dose** | | |
| Preparations | Ferric carboxymaltose (Ferinject); Ferric derisomaltose (Monoferric); Iron sucrose (Venofer). Note: BSH advises inadequate data for iron dextran (Cosmofer) in pregnancy. | |
| Dose | Weight-based: 500–1000 mg | |
| **Contraindications** | | |
| 1st Trimester | Yes | |
| Hypersensitivity | Yes | |
| Iron Overload | Not specified | |
| Hepatic Impairment | Yes | |
| Active Infections | Yes | |
| **F. Monitoring Post-Treatment** | | |
| **Parameter** | **Oral Iron** | **IV Iron** |
| Test | FBC (Hb) | Not specified |
| Timing | 2–3 weeks | Not specified |
| Expected Response | Demonstrable rise in Hb, with no specific numeric threshold specified | Not specified |
| **Abbreviations:** BSH, British Society for Haematology; FBC, full blood count; GI, gastrointestinal; Hb, haemoglobin; IV, intravenous; Y, yes; N, no. | | |

**Supplementary Table S3:** Local NHS Site Guidelines’ Indications for Oral Iron by Trimester

| **1st Trimester Indications** | **Guidelines (N, (%))** |
| --- | --- |
| Hb <105g/L | 1, (2) |
| Hb 80-105g/L | 4, (8) |
| Hb <110g/L | 33, (67) |
| Routine Iron Supplementation unless contraindicated | 3, (6) |
| **2^nd^ Trimester Indications** |  |
| Hb 80-105g/L | 4, (8) |
| Hb <105g/L | 33, (67) |
| **Other Indications** |  |
| Haemoglobinopathy and Low Ferritin | 8, (16) |
| Ferritin <30ug/L | 27, (55) |
| High Risk Women | 13, (27) |
| MCV < 100fl | 3, (6) |

*(*High Risk Women were typically defined as those with twin/multiple pregnancies, vegan/vegetarian diet, known history of iron deficiency or multiparity.)*

**Supplementary Figure S1:** Haemoglobin Thresholds Used by Local NHS Site Guidelines to Indicate IV Iron Treatment

##### The number of local NHS site guidelines recommending intravenous iron at each haemoglobin threshold. NHS = National Health Service.

**Supplementary Table S4:** Local NHS Site Recommendations for IV Iron Indications

| **Criteria for IV Iron** | **Guidelines (N, (%))** |
| --- | --- |
| **Serum Ferritin** | 6, (12) |
| <10ug/L | 1, (2) |
| <15ug/L | 1, (2) |
| <30ug/L | 3, (6) |
| <50ug/L | 1, (2) |
| **Late Gestation** | 30, (61) |
| Hb <90ug/L at >34 weeks | 3, (6) |
| Hb <105g/L at >34 weeks | 4, (8) |
| Hb <100g/L at >28 weeks | 7, (14) |
| Low Ferritin at >34 weeks | 1 (2) |
| Non-specific Urgent Correction | 18, (37) |
| **Other:** |  |
| Non-Compliance | 22, (45) |
| Intolerance | 43, (88) |
| Failure to Respond | 29, (59) |
| Malabsorption | 27, (55) |
| Significantly symptomatic | 3, (6) |

**Supplementary Table S5:** Local NHS Site Guidelines’ Definitions of Adequate Response to Oral Iron Treatment

| **Definition of Treatment Response** |  | **Guidelines (N, (%)** |
| --- | --- | --- |
| **Hb Increase** | **Week(s)** |  |
| Any Hb Increase | - | 16, (33) |
| 10-20g/L | 4 | 2, (4) |
| 10gL | 1 | 4, (8) |
| 10g/L | 2-4 | 5, (10) |
| 20g/L | 4-6 | 4, (8) |
| **Other** |  |  |
| Increase in RDW | - | 1, (2) |
| Not Mentioned | - | 17, (35) |
